# What are the economic costs of a poor work environment?

**DOI:** 10.5271/sjweh.4143

**Published:** 2024-03-01

**Authors:** Reiner Rugulies, Alex Burdorf

**Affiliations:** National Research Centre for the Working Environment (NFA) Department of Public Health and Department of Public Health, University of Copenhagen, Denmark. [email: rer@nfa.dk]; Erasmus Medical Center Rotterdam, Copenhagen, Denmark Rotterdam, The Netherlands. [email: a.burdorf@erasmusmc.nl]

At the *Scandinavian Journal of Work, Environment & Health*, it is our fundamental conviction that workers’ health is a value in itself. To put it simply, work must not be health-hazardous, and work must not make the worker neither physically nor mentally sick. In our minds, there is no need for any further rationale for healthy and safe work.

That said, it would be naïve to think that, in a bottom-line world, the bottom-line would not count with regard to work and health. It does count at individual, company, and societal level. At individual level, a worker may worry about reduced payment during sickness absence. At company level, the phrase “is there a business case?” is often heard. At societal level, all economic consequences, rather than a partial interest, are considered. Therefore, there is a broad need to know the magnitude of the economic loss that comes with a health-hazardous work environment and, vice-versa, the magnitude of the economic benefit that comes with improving the work environment. For example, when the World Health Organization (WHO) published in 2022 its landmark guidelines on mental health at work using a societal perspective (1), a lot of attention was paid to that WHO estimated the global economic costs of the most prevalent mental health conditions at USD1 trillion per year (2, 3).

There is, thus, a great demand for work environment economics, for analyses that quantify the economic costs of a poor work environment and the economic benefits of a good work environment. However, this demand is yet not being met by the research community. As van Dongen & van der Beek (4) delineated in an editorial in this Journal in May 2022, there are two types of work environment economic studies: (i) observational studies that examine the economic costs of work-related ill-health among workers, the so-called 'cost of illness' studies and (ii) economic evaluation studies that examine cost-effectiveness and return-on-investment of occupational health interventions. With regard to the latter, van Dongen & van der Beek point to a couple of recent promising evaluation studies (5, 6), however, they conclude that the methodological quality of economic evaluations of occupational health interventions leaves in general a lot to be desired (4).

With regard to the former, observational studies on the economic costs of work-related ill-health among workers, Russo et al (5) recently published in this Journal a cost-estimation model for work-related stress that was tested in two case studies in Italy – in healthcare and public administration. Costs were estimated as loss of productivity due to sickness absence attributable to work-related stress. The authors showed that across different work organizations, the proportion of sickness absence attributable to work-related stress varied strongly, and that methodological choices influenced the cost estimations largely.

In this issue of the Journal, Pedersen and colleagues (7) go a step further and present a paper that aims to estimate the effect of work-related stress on labor market costs at the societal level. Linking survey data from Denmark on indicators of self-reported work-related stress to register data on labor market affiliation, they first calculated the two-year prospective association between exposure to work stress and days of recurrent (being at work, sickness absence, unemployment, being temporarily out of the labor market for other reasons) and absorbing (retirement, disability pension, death) labor market states. Absorbing states occurred only rarely in their sample. Next, they calculated the costs incurred by the recurrent labor market states, ie, the costs associated with days lost due to all work absenteeism combined and work absenteeism stratified by sickness absence, unemployment, and being temporarily out of the labor market. Based on these calculations, the authors estimated an average annual work absenteeism loss per employee of €1903 for men and €3909 for women. For the Danish workforce, this corresponds to annual expenses of €305 million among men and €868 million among women, a total expense of €1.17 billion or 0.3% of the Danish gross domestic product in 2022.

The results of this article caused a lot of interest in the Danish media and among unions and employer organizations (8, 9). We commend the authors for their bold approach to estimate the costs of an assumed work-environment-induced health condition at the societal level, taking advantage of the excellent Danish health and labor market registers and using multi-state modeling that takes recurrence of events into consideration. There are, though, also important challenges in the article, relating both to the outcome and health condition under study.

Regarding the outcome, there are two major labor market costs to consider: (i) costs related to absenteeism, ie, costs due to not being at work, and (ii) costs related to loss of productivity, ie, costs due to being at work but with reduced work capacity. Pederson and colleagues limited their analyses to the former and did not analyze the latter. This is understandable, as absenteeism can be closely monitored in Danish national registers, whereas no national register is available for monitoring the productivity of workers. In terms of internal validity, it was therefore likely a wise decision by the authors to limit the analyses to an outcome where good data is available. However, as a consequence, an important part of the labor market costs of work-related stress – the loss in productivity among those who are present at work with reduced work capacity – could not be addressed in the article.

Regarding the health condition under study, the authors analyzed work-related stress. On the one hand, this is laudable, stress at work is topical and a major point of public debate in many countries, including Denmark. On the other hand, the authors could hardly have chosen a more difficult health condition. Stress is on many accounts an intricate, some would say, hopeless concept in research. Since the early days of stress research by Walter B Cannon (1871–1945) and Hans Selye (1907–1982), the concept of stress has been plagued with ambiguities (10, 11). Today, there is no lack of elaborated stress models (stimulus-based models, reaction-based models, the transactional model, the allostatic load model, the cognitive-activation theory of stress, to name just a few) (12–14). However, there is no common definition of stress, and there is no agreement on basic questions, such as if stress research should focus on the environmental conditions (often called “stressors”) that may cause cognitive, emotional, behavioral, and physiological changes in the individual (often called “stress reactions”) or if the focus should be on these stress reactions of the individual and how these different types of reactions are related to each other.

To make things further complicated, Pedersen and colleagues not only analyzed “stress”, but they analyzed “work-related stress”, thus, they based their analyses on the assumption that the stress condition reported by the participants were, to a considerable extent, caused by the work environment. To ensure the work-relatedness of their stress concept, Pedersen and colleagues combined three very different measures into one index. The three measures included (i) a kind of self-labeling method, where the definition of stress and the attribution to work or none-work related causes were solely at the discretion of the respondents; (ii) the measurement of a psychosocial work environment condition, job strain, that has been conceptualized as a stressor, ie, as an entity that is assumed to elicit stress reactions (15); and (iii) four items of Cohen’s Perceived Stress Scale (PSS-4), which is probably the most widely used questionnaire for assessing stress (16, 17), however, in a modified version that included references to work in three of the four items.

When taking a closer look at the three stress measures, one cannot help but to wonder whether the stress index by Pedersen and colleagues is rather a measure of poor psychosocial working conditions than a measure of stress. One of the three measures, job strain, obviously, is conceptualized as a measure of psychosocial working conditions, defined by the simultaneous exposure of high job demands and low job control (15). But also the modified PSS-4 items that asked, for example, about feeling that difficulties at work were piling up or feeling confident about handling problems at work, may have captured important parts of the psychosocial work environment. Thus, it could be argued that the article is first and foremost about the labor market costs of poor psychosocial working conditions.

To summarize, Pedersen and colleagues provided us with a thought-provoking paper on two hot but also very challenging topics in occupational health research, work-related stress and work environment economics. Both topics are likely strongly shaped by macro-level structural conditions that vary greatly between countries (18, 19). We look forward to further analyses and papers on these topics from other regions of the world.
